# Pioglitazone use increases risk of Alzheimer's disease in patients with type 2 diabetes receiving insulin

**DOI:** 10.1038/s41598-023-33674-2

**Published:** 2023-04-24

**Authors:** Hsin-Chung Lin, Chi-Hsiang Chung, Lih-Chyang Chen, Jui-Yang Wang, Chien-Chou Chen, Kuo-Yang Huang, Ming-Hang Tsai, Wu-Chien Chien, Hsin-An Lin

**Affiliations:** 1 Division of Clinical Pathology, Department of Pathology, Tri-Service General Hospital, National Defense Medical Center, Taipei City, 11490 Taiwan; 2 Graduate Institute of Pathology and Parasitology, National Defense Medical Center, Taipei City, 11490 Taiwan; 3 School of Public Health, National Defense Medical Center, Taipei City, 11490 Taiwan; 4 Taiwanese Injury Prevention and Safety Promotion Association, Taipei City, 11490 Taiwan; 5 Department of Medicine, Mackay Medical College, New Taipei City, 252 Taiwan; 6 Department of Family Medicine, Tri-Service General Hospital Songshan Branch, National Defense Medical Center, Taipei City, 10581 Taiwan; 7 Division of Nephrology, Department of Medicine, Tri-Service General Hospital Songshan Branch, National Defense Medical Center, Taipei City, 10581 Taiwan; 8 Department of Medicine, Tri-Service General Hospital Songshan Branch, National Defense Medical Center, Taipei City, 10581 Taiwan; 9 Department of Medical Research, Tri-Service General Hospital, National Defense Medical Center, No.161, Sec. 6, Minquan E. Rd., Neihu Dist., Taipei City, 11490 Taiwan; 10 Graduate Institute of Life Sciences, National Defense Medical Center, Taipei City, 11490 Taiwan; 11 Division of Infection, Department of Medicine, Tri-Service General Hospital Songshan Branch, National Defense Medical Center, No. 131, Jiankang Rd., Songshan District, Taipei City, 10581 Taiwan

**Keywords:** Neurological disorders, Alzheimer's disease

## Abstract

Pioglitazone is an insulin resistance inhibitor widely used as monotherapy or combined with metformin or insulin in treating type 2 diabetes mellitus (T2DM). This study further investigated the relationship between pioglitazone use and the risk of developing Alzheimer's disease (AD) in patients newly diagnosed with T2DM, and examined the potential impact of insulin use on this association. Data were extracted from the National Health Insurance Research Database (NHIRD) of Taiwan. Our data exhibited that the risk of developing AD in the pioglitazone group was 1.584-fold (aHR = 1.584, 95% CI 1.203–1.967, *p* < 0.05) higher than that in the non-pioglitazone controls. Compared to patients without both insulin and pioglitazone, higher cumulative risk of developing AD was found in patients receiving both insulin and pioglitazone (aHR = 2.004, 95% CI = 1.702–2.498), pioglitazone alone (aHR = 1.596, 95% CI = 1.398–1.803), and insulin alone (aHR = 1.365, 95% CI = 1.125–1.572), respectively (all *p* < 0.05). A similar observation also found in the evaluation the use of diabetic drugs with a cumulative defined daily dose (cDDD). No interaction between pioglitazone and major risk factors (comorbidities) of AD was observed. In conclusion, alternative drug therapies may be an effective strategy for reducing risk of developing AD in T2DM patients.

## Introduction

The incidence of diabetes mellitus (DM) in Taiwan has increased by 70% in the early twenty-first century, in line with the global trend of a 35% increase in the diabetes population^1^. DM is a major global public health issue, estimating 552 millions of people worldwide by 2030^2^. Notably, recent studies have linked diabetes with an increased risk of developing Alzheimer's disease (AD)^3,4^. In an observational study, type 2 diabetes mellitus (T2DM) was found to nearly double the risk of AD^5^, suggesting that diabetes may play a critical role in the development of AD pathogenesis.

The treatment of T2DM is generally aimed at normalizing hyperglycemia, lowering blood pressure, and correcting dyslipidemia. Metformin is the most commonly used first-line hypoglycemic agent in the world, and second-line oral hypoglycemic agents are aimed at potential insulin resistance^6–9^. Pioglitazone is an insulin resistance inhibitor that is used as monotherapy for T2DM, but it can also be used in combination with other diabetes medications, such as metformin or insulin^7,9^. In DM patients, clinical trials of pioglitazone as a single therapy or combined with metformin, insulin or sulfonylurea drugs showed that short-term and long-term glycemic control and lipid profiles are significantly improved^10^.

The mechanisms underlying the link between T2DM and AD are not fully understood, but research suggests that both cerebrovascular and non-cerebrovascular mechanisms may play a role^11^. Non-cerebrovascular mechanisms that may link T2DM with AD include hyperinsulinemia and advanced glycosylation end products. Insulin can cross the blood–brain barrier, and studies have shown that peripheral insulin infusion in the elderly lead to increased levels of the 42-amino-acid β-amyloid (Aβ42) in the cerebrospinal fluid^12^. Aβ42 is an alternative marker in the brain Aβ clearance, and an indirect marker of AD risk. Insulin in the brain has been shown to increase the deposition of Aβ and tau protein phosphorylation, which are key pathological features of AD^13^. However, to date, no study has evaluated associations between pioglitazone use and developing AD in patients with T2DM received insulin, although a recent study reports that pioglitazone reduces dementia risk in patients with T2DM^14^. The present study aimed to determine associations between pioglitazone use and developing AD in patients with newly diagnosed T2DM, and to evaluate the effect of insulin use on that association.

## Methods

### Data source

All data for the present study were extracted from the database of the universal National Health Insurance (NHI) program in Taiwan, which covers more than 99% of the Taiwanese population (23 million people), making it a nationally representative population^15^. The NHIRD contains comprehensive demographic and clinical data from outpatient and inpatient claims. All clinical diagnoses and procedures were recorded according to the International Classification of Diseases, 9th revision, Clinical Modification (ICD-9-CM) code.

### Ethical considerations

The study protocol, including study purpose, procedures involved, characteristics of data to be analyzed, access, security and management, and potential risks/benefits of this project, was approved by the Institutional Review Board of the Tri-Service General Hospital SongShan Branch (approval No. E202216021) and all methods were performed in accordance with the relevant guidelines and regulations of the Ministry of Health and Welfare (Taiwan). The requirement for informed consent was waived by the same IRB because all patient data are de-identified in the NHIRD, preserving the anonymity of included patients.

### Study design and sample

**Figure 1:**
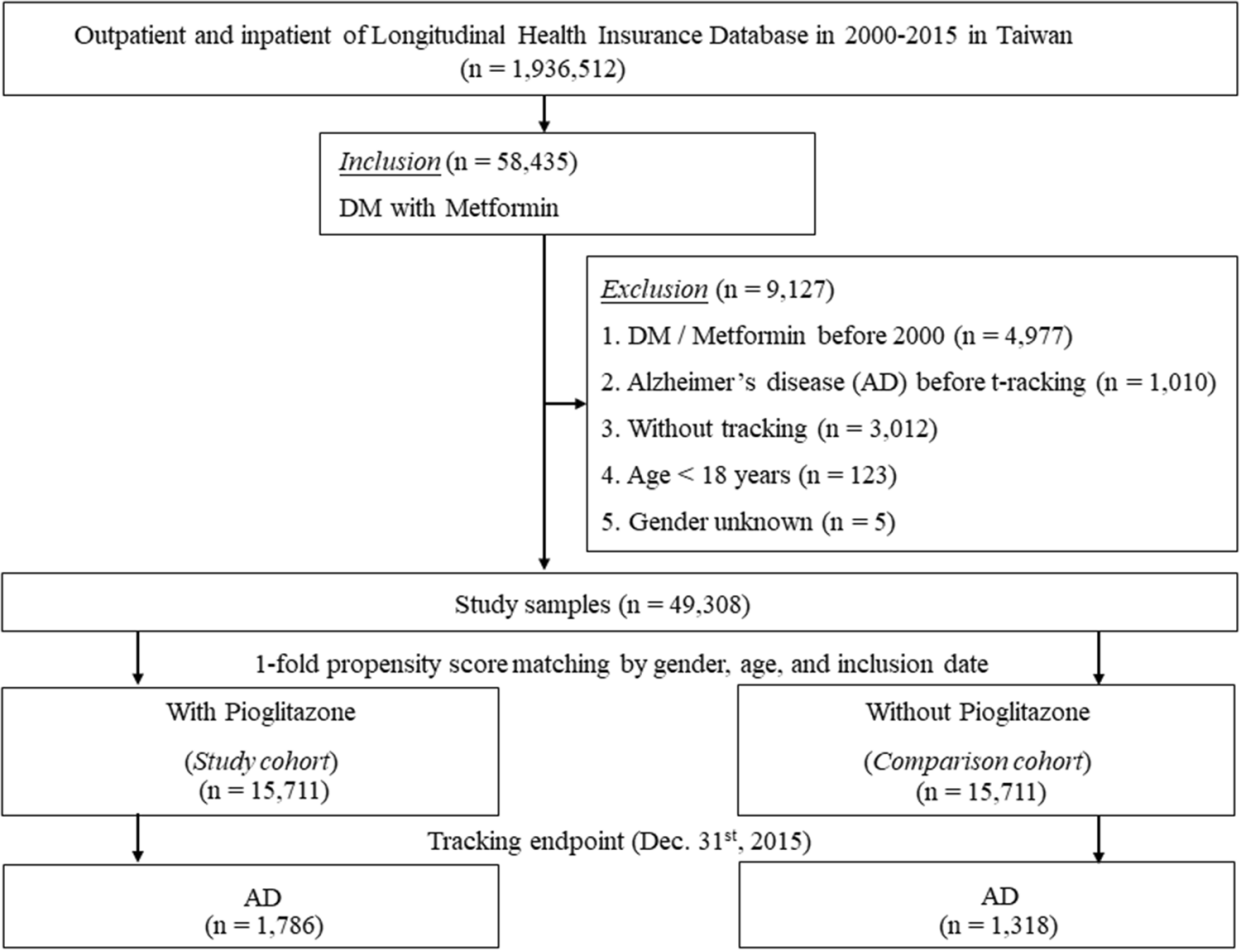
Flowchart for selection of study population.

This study used a retrospective cross-sectional cohort design. Figure 1 presents a flowchart indicating how the study population was selected. Medical records of approximately two million patients randomly selected from the NHIRD (1998–2015) were screened for eligibility. Eligible T2DM patients were identified by ICD-9-CM code 250, after patients with T1DM were excluded based on ICD-9-CM codes 250. × 1 and 250. × 3. Patients with T2DM were selected only if they were inpatients or had at least 3 outpatient visits. Patients diagnosed with T2DM and using metformin before the index date, who had AD before tracking, were under the age of 18, or had missing data on gender were also excluded. After exclusions, a cohort of T2DM patients receiving pioglitazone (n = 15,711) was established for comparison with a matched T2DM cohort not receiving pioglitazone. A total of 15,711 patients with T2DM who were not receiving pioglitazone was randomly selected as the reference cohort and matched according to gender, age, inclusion date, and occupation according to a 1:1 ratio of pioglitazone and non-pioglitazone patients. The estimation algorithm of propensity score matching was using logistic regression estimation and nearest neighbor matching with a tolerance of 0.20. The option for nearest neighbor was set as random matching order, non-replacement, no caliper, and 1 to 1 matching by gender, age, and inclusion date. In patients using pioglitazone, the index date was defined as three months after pioglitazone use. For those who never received pioglitazone, the index date was defined as three months after metformin use.

### Main outcomes

The principal outcome was AD (ICD-9-CM 331). Patients with AD were selected only if they were inpatients or had at least 3 outpatient visits. Patients receiving and not receiving pioglitazone were tracked from the index date to the onset of Alzheimer's disease or to the end of the study period (Dec. 31, 2015).

The independent variables were age, gender, insured premium, season, location of residence, urbanization level, hospital level, and the revised Charlson comorbidity index (CCI_R). CCI_R was defined as "CCI with diabetes mellitus removed". The Charlson Comorbidity Index (CCI), as adapted by Deyo et al.^16^, was used to assess the level of general comorbid conditions.

### Statistical analysis

The Chi-square test and Student's t-test were used to examine baseline demographic variables and comorbidities of pioglitazone and non-pioglitazone groups. Categorical variables were compared using the Chi-square test, and continuous variables were compared using the Student's *t*-test. Possible confounding variables (*p* < 0.05) among baseline variables listed in Table 1 were adjusted when subsequent statistical analyses were performed. The Omnibus test is a likelihood-ratio chi-square test of the current model versus the null model, and the significance value of less than 0.05 indicates that the current model outperforms the null model. The Omnibus test of our study exhibited a *P* < 0.001, indicating that it is a goodness-of-fit model. Moreover, the Schoenfeld residuals have a mean which is the true log hazard ratio under the proportional hazards assumption, and the average values of the Schoenfeld residuals over time can be interpreted as the time-varying log-hazard ratio. The significance value of Schoenfeld’s global test in this study showed a *P* = 0.901, indicating that it conforms the assumption of proportional hazards. Cox proportional hazards regression analysis was used to estimate the hazard ratios (HR) and 95% confidence intervals (CI) of AD in the pioglitazone cohort compared with those in the non-pioglitazone cohort. For causal analysis of competing risks, the Fine and Gray competing risk model with all-cause mortality as variant was used to confirm competing risk of mortality^17,18^. Kaplan–Meier analysis was used to measure the cumulative AD incidence for both study cohorts, and the log-rank test was used to evaluate differences between the two cumulative incidence curves. All statistical analyses were carried out using IBM SPSS statistical software version 22 for Windows (IBM Corp., Armonk, New York, USA). A p-value of less than 0.05 in 2-tailed tests was considered to be statistically significant.

**Table 1:** Characteristics of study population at the endpoint.

	Total (n = 31,422)	Pioglitazone (n = 15,711)	Non-pioglitazone (n = 15,711)	*p-*value
Alzheimer's disease, n (%)				< 0.001*
Without	28,318 (90.12)	13,925 (88.63)	14,393 (91.61)	
With	3104 (9.88)	1786 (11.37)	1318 (8.39)	
Gender, n (%)				0.999
Male	15,952 (50.77)	7976 (50.77)	7976 (50.77)	
Female	15,470 (49.23)	7735 (49.23)	7735 (49.23)	
Age (years), mean ± SD	59.82 ± 19.12	59.26 ± 18.95	60.37 ± 19.27	0.501
Age groups (years), n (%)				0.850
18–44	8381 (26.67)	4195 (26.70)	4186 (26.64)	
45–64	13,037 (41.49)	6537 (41.61)	6500 (41.37)	
≧ 65	10,004 (31.84)	4979 (31.69)	5025 (31.98)	
Insured premium (NT$), n (%)				< 0.001*
< 18,000	23,162 (73.71)	11,298 (71.91)	11,864 (75.51)	
18,000–34,999	5534 (17.61)	2703 (17.20)	2831 (18.02)	
≧ 35,000	2726 (8.68)	1710 (10.88)	1016 (6.47)	
CCI_R, mean (SD)	1.02 ± 1.01	1.03 ± 1.01	1.01 ± 1.00	0.078
Season, n (%)				0.661
Spring	7582 (24.13)	3796 (24.16)	3786 (24.10)	
Summer	8353 (26.58)	4165 (26.51)	4188 (26.66)	
Autumn	7907 (25.16)	3995 (25.43)	3912 (24.90)	
Winter	7580 (24.12)	3755 (23.90)	3825 (24.35)	
Location, n (%)				0.609
Northern Taiwan	9163 (29.16)	4596 (29.25)	4567 (29.07)	
Middle Taiwan	8109 (25.81)	4087 (26.01)	4022 (25.60)	
Southern Taiwan	8690 (27.66)	4331 (27.57)	4359 (27.74)	
Eastern Taiwan	3952 (12.58)	1934 (12.31)	2018 (12.84)	
Outlets islands	1508 (4.80)	763 (4.86)	745 (4.74)	
Urbanization level, n (%)				0.623
1 (The highest)	8990 (28.61)	4502 (28.66)	4488 (28.57)	
2	10,222 (32.53)	5096 (32.44)	5126 (32.63)	
3	5599 (17.82)	2768 (17.62)	2831 (18.02)	
4 (The lowest)	6611 (21.04)	3345 (21.29)	3266 (20.79)	
Level of care, n (%)				0.402
Hospital center	17,790 (56.62)	8923 (56.79)	8867 (56.44)	
Regional hospital	8127 (25.86)	4013 (25.54)	4114 (26.19)	
Local hospital	5505 (17.52)	2775 (17.66)	2730 (17.38)	

CCI_R, Revised Charlson comorbidity index (CCI with DM removed); SD, standard deviation. *p < 0.05.

## Results

### Patients’ baseline characteristics

A total of 31,422 adult patients with T2DM aged over 18 years were included, including 15,711 patients using pioglitazone and 15,711 patients not using pioglitazone (Fig. 1 and Table 1). No significant differences were found in age, gender, and CCI_R between the two cohorts at baseline or endpoint (all *p* > 0.05, Supplementary Table S1 and Table 1). However, significant differences were found between the groups in baseline salary-based insured premium, location of residence, and urbanization level (all *p* < 0.05, Supplementary Table S1).

### Associations between patients’ characteristics and incidence of Alzheimer's disease

The incidence of AD significantly increased in patients with pioglitazone use compared with that in patients without pioglitazone use (*p* < 0.05, Table 1). Table 1 shows results of Cox regression analysis adjusted for possible confounding variables, indicating that the incidence of AD in T2DM patients with pioglitazone use was approximately 1.584-fold higher (adjusted HR (aHR) = 1.584, 95% CI 1.203–1.967) than that in T2DM patients without pioglitazone use (*p* < 0.05, Table 2). In addition, high salary-based insured premium, high urbanization level, and high hospital level of care also was associated with incident AD (all *p* < 0.05, Table 2). After stratifying by the variables listed in Table 2 and analyzing using Cox regression adjusted for these variables, each subgroup among pioglitazone patients had a higher risk of AD than did subgroups in non-pioglitazone patients (all *p* < 0.05, Supplementary Table S2). Moreover, these stratifying variables were not significantly affected the risk of pioglitazone-related AD (all *p* > 0.05, Supplementary Table S2), suggesting that there may be no interactions between these variables and pioglitazone-related AD.

**Table 2:** Cox-regression analysis of factors associated with Alzheimer's disease (n = 31,422).

Variables	HR (95%CI)	*p*-value	aHR (95%CI)	*p*-value
Pioglitazone				
Without	Reference		Reference	
With	1.867 (1.579, 2.010)	< 0.001*	1.584 (1.203, 1.967)	< 0.001*
Gender				
Male	1.420 (1.145, 1.786)	< 0.001*	1.350 (1.107, 1.722)	< 0.001*
Female	Reference		Reference	
Age groups (years)				
18–44	Reference		Reference	
45–64	1.330 (1.096, 1.761)	0.001*	1.268 (1.074, 1.588)	0.015*
≧ 65	1.567 (1.134, 1.894)	< 0.001*	1.339 (1.088, 1.675)	0.002*
Insured premium (NT$)				
< 18,000	Reference		Reference	
18,000–34,999	0.952 (0.728, 1.154)	0.379	0.979 (0.799, 1.246)	0.427
≧ 35,000	0.757 (0.512, 1.089)	0.242	0.880 (0.672, 1.075)	0.198
CCI_R	1.335 (1.124, 1.555)	< 0.001*	1.201 (1.086, 1.428)	0.007*
Season				
Spring	Reference		Reference	
Summer	1.106 (0.673, 1.445)	0.522	1.084 (0.657, 1.420)	0.622
Autumn	0.986 (0.526, 1.368)	0.458	0.979 (0.511, 1.324)	0.517
Winter	1.274 (0.707, 1.497)	0.617	1.201 (0.699, 1.486)	0.666
Location				
Northern Taiwan	Reference			
Middle Taiwan	0.875 (0.559, 1.630)	0.543		
Southern Taiwan	0.986 (0.608, 1.842)	0.228		
Eastern Taiwan	0.771 (0.456, 1.511)	0.604		
Outlets islands	3.010 (0.078, 59.975)	0.972		
Urbanization level				
1 (The highest)	1.578 (1.248, 1.806)	< 0.001*	1.342 (1.201, 1.786)	< 0.001*
2	1.566 (1.231, 1.801)	< 0.001*	1.331 (1.199, 1.772)	< 0.001*
3	1.359 (1.124, 1.508)	< 0.001*	1.206 (1.099, 1.484)	0.001*
4 (The lowest)	Reference		Reference	
Level of care				
Medical center	2.296 (1.811, 2.635)	< 0.001*	2.010 (1.662, 2.448)	< 0.001*
Regional hospital	1.864 (2.249, 0.024)	< 0.001*	1.692 (1.381, 2.007)	< 0.001*
Local hospital	Reference		Reference	

aHR, adjusted hazard ratio (Adjusted variables listed in the table); CI, confidence interval; DM, diabetes mellitus; CCI_R, Revised Charlson comorbidity index (CCI with DM removed). *p < 0.05.

### Pioglitazone is a risk factor for Alzheimer's disease, while combined pioglitazone and insulin led to an increased risk of Alzheimer's disease

**Figure 2:**
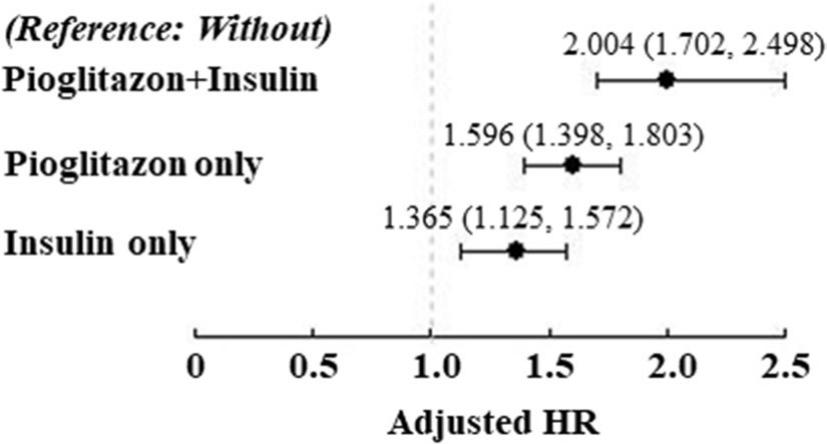
Cox regression analysis for risk of developing AD and T2DM patients receiving both insulin and pioglitazone.

In Cox regression analysis, compared to T2DM patients without both pioglitazone and insulin, T2DM patients receiving both pioglitazone and insulin had a higher risk of developing AD (aHR = 2.004, 95% CI = 1.702–2.498, *p* < 0.05, Fig. 2 and Supplementary Table S3), T2DM patients receiving pioglitazone alone had a higher risk of developing AD (aHR = 1.596, 95% CI = 1.398–1.803, *p* < 0.05, Fig. 2), and T2DM patients receiving insulin alone had a higher risk of developing AD (aHR = 1.365, 95% CI = 1.125–1.572, *p* < 0.05, Fig. 2 and Supplementary Table S3). T2DM patients received both pioglitazone and insulin had a higher risk of developing AD than that in T2DM patients received insulin alone (aHR = 1.469, 95% CI = 1.103–1.787, *p* < 0.05, Supplementary Table S3) or pioglitazone alone (aHR = 1.296, 95% CI = 1.030–1.498, *p* < 0.05, Supplementary Table S3), respectively.

Furthermore, a similar observation also found in the evaluation the use of diabetic drugs with a cumulative defined daily dose (cDDD) (Table 3), with risk of developing AD in each cDDD subgroup as the following order: pioglitazone plus insulin group > pioglitazone alone group > insulin alone group. In addition, patients receiving a higher cumulative defined daily dose (cDDD) of pioglitazone and insulin had a higher risk of developing AD than patients using a lower cDDD of pioglitazone and insulin (aHR = 1.736, 1.852, and 2.365 for cDDD < 258, 258–713 and ≧ 714, respectively; all *p* < 0.05, Table 3). Similarly, among patients who received pioglitazone alone, patients with a higher cDDD of pioglitazone had a higher risk of developing AD than patients with a lower cDDD of pioglitazone (aHR = 1.368, 1.484 and 1.836 for cDDD < 258, 258–713 and ≧714, respectively; all *p* < 0.05, Table 3). Among patients who received insulin alone, patients with a higher cDDD of insulin had a higher risk of developing AD than those with a lower cDDD of insulin (aHR = 1.279, 1.288 and 1.530 for cDDD < 258, 258–713 and ≧ 714, respectively; all *p* < 0.05, Table 3).

**Table 3:** Factors of AD among different cDDD by Cox regression with/without Fine and Gray's competing risk model (n = 31,422).

Groups, cDDD	aHR (95% CI)	*p*-value
Pioglitazone and insulin		
cDDD: < 258	1.736 (1.474, 2.164)	< 0.001*
cDDD: 258–713	1.852 (1.573, 2.309)	< 0.001*
cDDD: ≧ 714	2.365 (2.008, 2.947)	< 0.001*
Pioglitazone alone		
cDDD: < 258	1.368 (1.198, 1.545)	< 0.001*
cDDD: 258–713	1.484 (1.300, 1.676)	< 0.001*
cDDD: ≧714	1.836 (1.608, 2.074)	< 0.001*
Insulin alone		
cDDD: < 258	1.279 (1.054, 1.473)	0.001*
cDDD: 258–713	1.288 (1.062, 1.483)	< 0.001*
cDDD: ≧714	1.530 (1.261, 1.762)	< 0.001*
Without	Reference	

AD, Alzheimer's disease; cDDD, cumulative defined daily dose; aHR, adjusted hazard ratio (adjusted for the variables listed in Table 2); CI, confidence interval. *p < 0.05.

**Figure 3:**
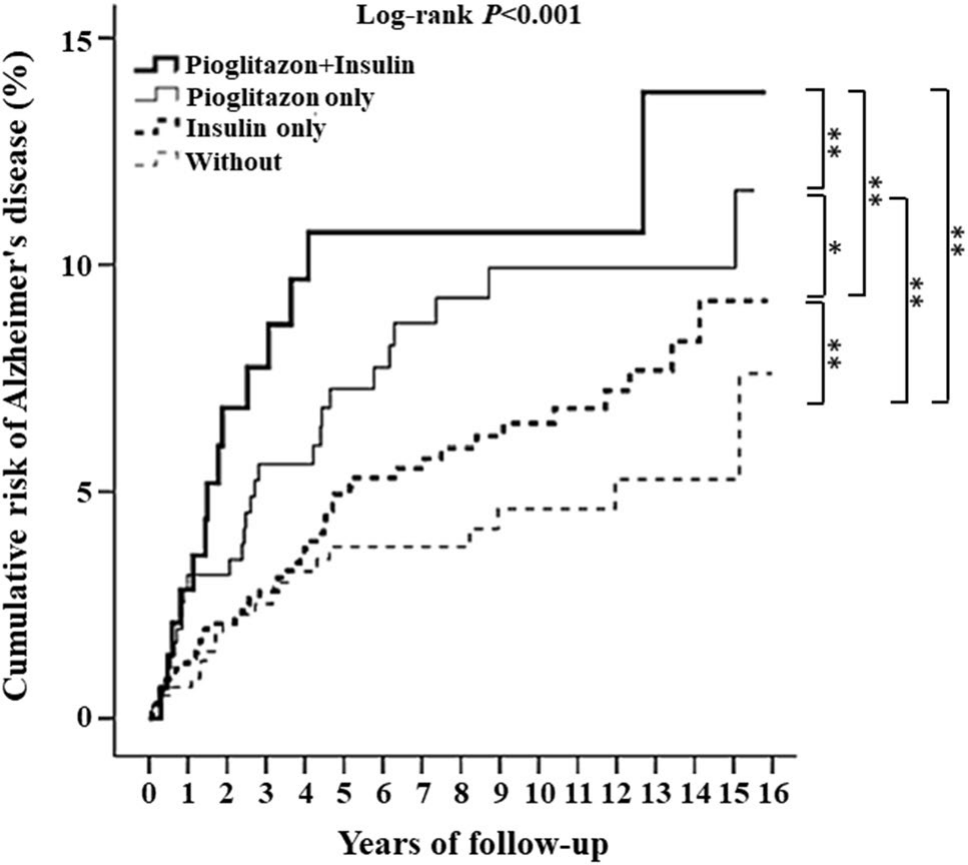
Kaplan–Meier plot for cumulative incidence of AD in T2DM patients without or with pioglitazone plus insulin, pioglitazone alone, or insulin alone.

Subsequently, Kaplan–Meier curve analysis disclosed that patients receiving pioglitazone and insulin had a significantly higher cumulative risk of developing AD than those using pioglitazone alone or insulin alone (all *p* < 0.001, Fig. 3). Among patients not receiving insulin, T2DM patients with pioglitazone also had a significantly higher cumulative risk of developing AD than those not using pioglitazone (*p* < 0.001, Fig. 3). Moreover, patients using pioglitazone alone also had a significantly higher cumulative risk of developing AD than those using insulin alone (*p* < 0.05, Fig. 3).

## Discussion

The present analysis of pioglitazone treatment and risk of developing AD in patients with T2DM yielded two new key findings: the pioglitazone treatment was an independent predictive factor for the occurrence of AD and concomitant insulin treatment enhanced the risk of pioglitazone-related AD. Patients with increased odds of developing AD were of older age and male sex, had high salary-based insured premiums, resided in areas of high urbanization, and received higher levels of hospital care.

Previous studies have shown that T2DM is associated with an increased risk of AD^5^. This association may be due, in part, to a newly recognized form of brain insulin resistance that is also associated with AD^19,20^. The increase in AD prevalence in Western societies reflects a cohort effect, with those born later in life having a lower risk of developing AD than those born earlier in the last century^4^. According to the patient's age of onset, AD is classified as early-onset AD (< 65 years old) accounted for 1%-5% of all cases, and late-onset AD (≥ 65 years old) accounted for more than 95% of all cases^4^. In addition, males and comorbidities such as T2DM, hypertension, obesity, dyslipidemia, and metabolic syndrome have been identified as possible risk factors for the development of AD^4,21^. Similarly, the present study demonstrated that age, male sex, and CCI-R (defined as “CCI with diabetes mellitus removed”) were independent risk factors for developing AD. Expanding these findings, the present study showed further that higher salary-based insured premium, higher urbanization, and higher hospital care levels were associated with an increased risk of developing AD. A recent study supported these findings, indicating that areas with high median annual family income were associated with higher odds of developing dementia^22^.

In the present study, after adjusting for the above confounding factors, T2DM patients receiving insulin treatment had an increased risk of developing AD compared with the risk in patients not receiving insulin and pioglitazone treatment. Moreover, the cumulative dose of insulin was also associated with an even more significant increase in risk. A previous study reports that insulin crosses the blood–brain barrier and that peripheral insulin infusion in normal adult increases Aβ42 level in the cerebrospinal fluid, which makes it a surrogate marker of Aβ clearance and an indirect marker of brain and AD risk^12^. Other evidence also shows that soluble Aβ oligomer (AβOs) lead to the significant loss of insulin receptor (IRs) in neuronal process membrane^23^. AβOs are small aggregates of beta-amyloid proteins that accumulate in the brain of AD patients and considered to be an effective synaptotoxin^24,25^. Oligomers specifically attach to the synapses of specific neurons and act as pathogenic ligands^26,27^. Recent studies have suggested that the conjugation of AβOs can induce AD-like pathologies, including neuronal tau protein hyperphosphorylation^28^, oxidative stress^29,30^, synaptic degeneration and loss^30,31^, and inhibition of synaptic plasticity^32^, which may be central to insulin treatment involved in the pathogenesis of AD in T2DM patients.

A recent study reports that pioglitazone reduces dementia (defined as senile dementia, vascular dementia, frontotemporal dementia, dementia associated with brain degeneration, senile degeneration-related dementia, AD [ICD-9 CM 331], cerebral lipid-related dementia) risk in patients with T2DM^14^. In contrast, our data showed that T2DM patients receiving pioglitazone alone had a higher risk of developing AD (ICD-9 CM 331) than those receiving or not receiving insulin alone. In particular, T2DM patients with pioglitazone and insulin had a higher risk of developing AD than those receiving pioglitazone alone or insulin alone. Moreover, the cumulative dose of pioglitazone plus insulin was associated with an even more significant increase in risk. As we stated earlier, pioglitazone is an insulin resistance inhibitor widely used as monotherapy or combined with metformin or insulin. There is also growing evidence that insulin resistance develops in the brain of patients with AD^19^. Insulin and IR levels in AD are low in the brain and insulin signaling damage was recorded in both autopsy and AD animal models^20,33,34^. Insulin signaling of brain is particularly important for learning and memory^35,36^, and that insulin resistance may lead to cognitive deficits in AD. Because the pathological features such as impaired insulin signal seem to be shared by T2DM patients who use pioglitazone alone and patients with AD, we speculated that mechanisms analogous to those that account for peripheral insulin resistance in T2DM may underlie impaired brain insulin signalling in pioglitazone-induced development of AD. Compared to T2DM patients who received pioglitazone, both insulin-induced Aβ accumulation in the brain and peripheral insulin resistance-impaired brain insulin signalling are possible mechanisms in T2DM patients who are receiving both pioglitazone and insulin.

Understanding the molecular mechanism of brain insulin signal impairment may clarify a new method to counteract the damage of AD neurons. Results reported here suggest that insulin signalling may be disrupted in AD brains by mechanisms similar to those leading to insulin resistance in T2DM patients who received pioglitazone treatment. Aberrant activation of the JNK/TNF-α pathway has been shown to occur in neurons exposed to AβOs both in vivo and in vitro. This abnormal activation leads to the serine phosphorylation of IRS-1, a key insulin receptor substrate^37^, which blocks downstream insulin signalling and can trigger peripheral insulin resistance in individuals with DM^38^. Recent study reports significantly that exendin-4, a new drug for DM, activates the common pathway of insulin signal transduction by stimulating glucagon-like peptide-1 (GLP-1) receptor, thus blocking the damage of insulin signal transduction in hippocampal neurons, reversing insulin pathology and improving the cognitive ability of mice^37^. Thus, we suggest that stimulation of GLP-1 receptors may represent a promising new approach to prevent disruption of brain insulin signalling in patients with AD or T2DM.

Cognitive, social and intellectual activity with higher education and professional achievement has been shown to reduce the risk of cognitive decline and dementia by increasing cognitive decline, which are the brain's ability to resist the effects of neurological injury^39^. A meta-analysis has shown that engaging in mentally stimulating activities can reduce the risk of developing AD^40^. Approximately 19% of AD cases worldwide can be attributed to low educational attainment, making it the highest risk factor for the proportion of AD cases^40^. Helping to build cognitive reserve so individuals can continue to function at normal levels, despite neurodegenerative changes that appear to play a large role in disease onset. Recently, the beneficial effects of bilingualism on brain reserves have been highlighted as beneficial to AD risk and cognition^41,42^. According to these findings, we suggest that promoting cognitive reserve may be another beneficial strategy for preventing neurodegeneration in patients with T2DM.

### Limitations

This study has several limitations. First, certain risk factors for AD, including educational level, physical function and other well known risk factors, are not included in the analysis due to lack of available data in the NHIRD. Although we used salary-based insured premium as a proxy for individual educational level, used the revised Charlson comorbidity index (included general comorbid conditions) as a proxy for other well-known risk factors (such as hypertension, metabolic syndrome, dyslipidemia and smoking) and used the hospital care level as a proxy for physical function, residual confounding bias is still possible. In addition, the behavioral changes of the attending physicians' drug prescription may be affected by comorbidities, such as cardiovascular diseases or kidney disease. To address this concern, Cox regression analysis adjusted for these possible confounding factors. Second, we relied on the physician-recorded diagnosis in the NHIRD medical claims to select cases with T2DM or AD, which may result in disease misclassification. To address this concern, patients with T2DM were selected only if they were inpatients or had at least 3 outpatient visits or they were diagnosed with T2DM who used metformin before the index date. Patients with AD were selected only if they were inpatients or had at least 3 outpatients visits. Third, given that the diagnostic procedures of AD can vary across medical resources and medical care levels, we adjusted for the hospitals and clinics in each township and the level of urbanization to reduce the differences in medical resources and medical care that may result in unequal opportunity to be diagnosed as having AD. Forth, because pioglitazone is generally used as a third-line antidiabetic drug and insulin is always the last resort. Those who used these drugs were prone to have later stages of diabetes. Moreover, other oral anti-diabetic drugs, including acarbose^43^, rosiglitazone^44^, vildagliptin^45^ and metformin^46^, may also affect the risk of developing dementia. However, the present study may not have directly evaluated the effects of anti-diabetic drugs on the development of AD. To address these concerns, we also evaluated the use of diabetic drugs with a cumulative defined daily dose (cDDD). Our data exhibited that patients receiving a higher cDDD of pioglitazone/insulin, pioglitazone alone, or insulin alone had higher risk of developing AD than those using a lower cDDD of pioglitazone/insulin, pioglitazone alone, or insulin alone, respectively.

## Conclusions

Higher risk of developing AD is associated with pioglitazone use and insulin therapy in T2DM patients. Managing the use of pioglitazone alone and the combination of pioglitazone and insulin may have a potentially positive effect on decreasing the risk of developing AD in patients with T2DM. Potential pathways via possible mediators of risk factors for developing AD should be further examined in future studies.

## Supplementary Information

Supplementary Information.

## Data Availability

The datasets used and/or analysed during the current study are available from the corresponding author on request.
