# Supplementary material for: Pioglitazone use increases risk of Alzheimer's disease in patients with type 2 diabetes receiving insulin
Source: Sci Rep. 2023 Apr 24;13:6625. doi: 10.1038/s41598-023-33674-2 (PMC10126143; doi:10.1038/s41598-023-33674-2)
Supplement: Supplementary file 1 — Supplementary Information. [file 41598_2023_33674_MOESM1_ESM.docx]

**Supplementary Material**

**Pioglitazone use increases risk of Alzheimer's disease in patients with type 2 diabetes receiving insulin**

Hsin-Chung Lin, MD, PhD ^1,2^, Chi-Hsiang Chung, PhD ^3,4^, Lih-Chyang Chen, PhD ^5^, Jui-Yang Wang, MD ^6^, Chien-Chou Chen, MD ^7^, Kuo-Yang Huang, PhD ^2^, Ming-Hang Tsai, MD ^8^, Wu-Chien Chien, PhD ^3,9,10,*^, Hsin-An Lin, MD ^11,*^

***Correspondemce.**

Hsin-An Lin

Division of Infection, Department of Medicine, Tri-Service General Hospital SongShan Branch, National Defense Medical Center, No. 131, Jiankang Rd., Songshan District, Taipei City 10581, Taiwan

Tel.: 886 2 27642151#672134,

shinean0928@gmail.com

Wu-Chien Chien

Department of Medical Research, Tri-Service General Hospital, National Defense Medical Center, No.161, Sec. 6, Minquan E. Rd., Neihu Dist., Taipei City 11490, Taiwan

Tel.: 02-87923311 # 19189,

chienwu@mail.ndmctsgh.edu.tw

| **Supplementary Table 1. Baseline characteristics of study population by group.** | | | | | | | | | |
| --- | --- | --- | --- | --- | --- | --- | --- | --- | --- |
|  | **Total**  **(n=31,422)** | | **Pioglitazone**  **(n=15, 711)** | | | **Non-pioglitazone**  **(n=15,711)** | | | ***p-*value** |
| **Gender**, n (%) |  |  | |  |  | |  |  | 0.999 |
| Male | 15,952 (50.77) | | 15,952 (50.77) | | | 7,976 (50.77) | | |  |
| Female | 15,470 (49.23) | | 15,470 (49.23) | | | 7,735 (49.23) | | |  |
| **Age (years)**, mean ± SD | 52.04 ± 18.80 | | 52.11 ± 18.86 | | | 51.97 ± 18.74 | | | 0.501 |
| **Age groups (years)**, n (%) |  |  | |  |  | |  |  | 0.999 |
| 18-44 | 8,402 (26.74) | | 4,201 (26.74) | | | 4,201 (26.74) | | |  |
| 45-64 | 13,138 (41.81) | | 6,569 (41.81) | | | 6,569 (41.81) | | |  |
| ≧65 | 9,882 (31.45) | | 4,941 (31.45) | | | 4,941 (31.45) | | |  |
| **Insured premium (NT$)**, n (%) |  |  | |  |  | |  |  | <0.001* |
| <18,000 | 23,162 (73.71) | | 11,298 (71.91) | | | 11,864 (75.51) | | |  |
| 18,000-34,999 | 5,534 (17.61) | | 2,703 (17.20) | | | 2,831 (18.02) | | |  |
| ≧35,000 | 2,726 (8.68) | | 1,710 (10.88) | | | 1,016 (6.47) | | |  |
| **CCI_R**, mean (SD) | 1.01 ± 1.00 | | 1.01 ± 1.01 | | | 1.00 ± 0.99 | | | 0.376 |
| **Season**, n (%) |  |  | |  |  | |  |  | 0.999 |
| Spring (Mar-May) | 7,622 (24.26) | | 3,811 (24.26) | | | 3,811 (24.26) | | |  |
| Summer (Jun-Aug) | 8,250 (26.26) | | 4,125 (26.26) | | | 4,125 (26.26) | | |  |
| Autumn (Sep-Nov) | 7,972 (25.37) | | 3,986 (25.37) | | | 3,986 (25.37) | | |  |
| Winter (Dec-Feb) | 7,578 (24.12) | | 3,789 (24.12) | | | 3,789 (24.12) | | |  |
| **Location**, n (%) |  |  | |  |  | |  |  | <0.001* |
| Northern Taiwan | 8,894 (28.31) | | 4,582 (29.16) | | | 4,312 (27.45) | | |  |
| Middle Taiwan | 8,160 (25.97) | | 4,043 (25.73) | | | 4,117 (26.20) | | |  |
| Southern Taiwan | 8,521 (27.12) | | 4,312 (27.45) | | | 4,209 (26.79) | | |  |
| Eastern Taiwan | 3,999 (12.73) | | 1,986 (12.64) | | | 2,013 (12.81) | | |  |
| Outlets islands | 1,848 (5.88) | | 788 (5.02) | | | 1,060 (6.75) | | |  |
| **Urbanization level**, n (%) |  |  | |  |  | |  |  | <0.001* |
| 1 (The highest) | 8,929 (28.42) | | 4,426 (28.17) | | | 4,503 (28.66) | | |  |
| 2 | 10,081 (32.08) | | 5,093 (32.42) | | | 4,988 (31.75) | | |  |
| 3 | 5,173 (16.46) | | 2,711 (17.26) | | | 2,462 (15.67) | | |  |
| 4 (The lowest) | 7,239 (23.04) | | 3,481 (22.16) | | | 3,758 (23.92) | | |  |
| **Level of care**, n (%) |  |  | |  |  | |  |  | 0.743 |
| Medical center | 17,911 (57.00) | | 8,986 (57.20) | | | 8,925 (56.81) | | |  |
| Regional hospital | 7,997 (25.45) | | 3,990 (25.40) | | | 4,007 (25.50) | | |  |
| Local hospital | 5,514 (17.55) | | 2,735 (17.41) | | | 2,779 (17.69) | | |  |
| CCI_R, Revised Charlson comorbidity index (CCI with DM removed); SD, standard deviation. *P:* Chi-square / Fisher exact test for categorical variables and t-test for continuous variables **p*<0.05. | | | | | | | | | |

**Supplementary Table 2. Cox-regression analysis of pioglitazone and risk of Alzheimer's disease stratified by patients’ characteristics (n = 31,422).**

|  | **Pioglitazone *vs.* Non-pioglitazone** | | ***p*-value for**  **interaction** |
| --- | --- | --- | --- |
|  | **aHR (95%CI)** | ***p*-value** |  |
| **Gender** |  |  | 0.925 |
| Male | 1.621 (1.231, 2.013) | <0.001* |  |
| Female | 1.542 (1.171, 1.915) | <0.001* |  |
| **Age groups (years)** |  |  | 0.806 |
| 18-44 | 1.565 (1.189, 1.944) | <0.001* |  |
| 45-64 | 1.575 (1.196, 1.956) | <0.001* |  |
| ≧65 | 1.611 (1.224, 2.001) | <0.001* |  |
| **Insured premium (NT$)** |  |  | 0.389 |
| <18,000 | 1.677 (1.273, 2.082) | <0.001* |  |
| 18,000-34,999 | 1.452 (1.103, 1.804) | <0.001 |  |
| ≧35,000 | 1.344 (1.021, 1.669) | 0.030* |  |
| **HTN** |  |  | 0.837 |
| Without | 1.524 (1.158, 1.893) | <0.001* |  |
| With | 1.662 (1.262, 2.064) | <0.001* |  |
| **Hyperlipidemia** |  |  | 0.981 |
| Without | 1.543 (1.172, 1.916) | <0.001* |  |
| With | 1.599 (1.214, 1.985) | <0.001* |  |
| **CKD** |  |  | 0.892 |
| Without | 1.482 (1.125, 1.840) | <0.001* |  |
| With | 1.648 (1.252, 2.047) | <0.001* |  |
| **Season** |  |  | 0.730 |
| Spring | 1.550 (1.177, 1.925) | <0.001* |  |
| Summer | 1.582 (1.202, 1.965) | <0.001* |  |
| Autumn | 1.543 (1.172, 1.916) | <0.001* |  |
| Winter | 1.665 (1.264, 2.067) | <0.001* |  |
| **Urbanization level** |  |  | 0.237 |
| 1 (The highest) | 1.671 (1.269, 2.075) | <0.001* |  |
| 2 | 1.633 (1.240, 2.028) | <0.001* |  |
| 3 | 1.427 (1.083, 1.771) | 0.008* |  |
| 4 (The lowest) | 1.340 (1.017, 1.663) | 0.041* |  |
| **Level of care** |  |  | 0.084 |
| Hospital center | 1.863 (1.415, 2.313) | <0.001* |  |
| Regional hospital | 1.410 (1.071, 1.751) | 0.013* |  |
| Local hospital | 1.341 (1.018, 1.665) | 0.042* |  |

aHR, adjusted hazard ratio; CI, confidence interval. Adjusted for the variables listed in Table. **p*<0.05

| **Supplementary Table 3. Cox-regression analysis of pioglitazone/insulin and risk of Alzheimer's disease (n = 31,422).** | | | | | | |
| --- | --- | --- | --- | --- | --- | --- |
|  | **aHR (95% CI)** | ***p-*value** | **aHR (95% CI)** | ***p-*value** | **aHR (95% CI)** | ***p-*value** |
| **Pioglitazone and insulin** | 2.004 (1.702, 2.498) | <0.001* | 1.296 (1.030, 1.498) | 0.021* | 1.469 (1.103, 1.787) | <0.001* |
| **Pioglitazone alone** | 1.596 (1.398, 1.803) | <0.001* | Reference |  | － |  |
| **Insulin alone** | 1.365 (1.125, 1.572) | <0.001* | － |  | Reference |  |
| **Without pioglitazone and insulin** | Reference |  | － |  | － |  |
| aHR, adjusted hazard ratio (adjusted for the variables listed in Table 2); CI, confidence interval. **p*<0.05. | | | | | | |
